# Pyrophosphate of Iron

**Published:** 1862-03

**Authors:** 


### PYROPHOSPHATE OF IRON.

Extracts from Prof. CHAPMAN'S Paper, on the History, Preparation and Therapeutical uses of the Citro-Ammoniacal Pyrophosphate of Iron, name in brief Pyrophosphate of Iron, in the *Boston Medical and Surgical Journal.*

The pyrophosphate of iron, a white and tasteless powder, resembling prepared chalk, is obtained as a gelatinous precipitate in the reaction between the pyrophosphate of soda and the tersulphate of iron in solution. A given proportion of citric acid in solution is neutralized by liquor ammoniæ, as shown by test paper, when phyrophosphate is added, and the liquid boiled until the salt is dissolved. We now have the citro-ammoniacal pyrophosphate of iron in solution; from which we may obtain the solid salt by evaporating to a thick consistency, and then spreading the product on large plates of glass. It takes the form of lamellæ of greater or less thickness; these, when thin, are flaky, brittle, and of a yellowish green color, but when more massive are of a duller and deeper green, and of a resinous appearance. This salt, which has a slightly saline taste, may be made into pills, or dissolved in water in any proportion, by the aid of heat. The solution does not require any addition to disguise it, as the saline taste is trivial and not unpleasant, though sugar and a flavoring ingredient may be added to suit the caprices of patients. Syrup completely conceals the iron and renders the preparation tasteless. This may be made of any desired strength. I usually give from three to five grains of the pyrophosphate three or four times a day.

The solid salt contains its ingredients in the following proportions to the hundred parts :—

| | |
|---|---|
| Pyrophosphate of iron (anhydrous) | 48.8 |
| Citrate of ammonia (neutral) | 34.66 |
| Water in combination | 16.54 |
| Total, | 100.00* |

The 48.8 parts of the pyrophosphate contain 7.27 of metalic iron. It is a sesqui-salt, represented by the formula 2 $Fe^2 O^3$, $3PO^5$, and formed by the reaction between three equivalents of the pyrophosphate of soda 3 (2 NaO, $PO^5$) and two of the tersulphate of iron 2 ($Fe^2 O^3$, 3 $SO^3$).

*See American Journal of Pharmacy, January, 1861, p. 39.

The citro-ammoniacal pyrophosphate of iron affords certain marked advantages over the preparations of iron hitherto in use. Its tastelessness, in solution with sugar, and elegant appearance, in our day, when the nauseous doses of the older practitioners will not be tolerated, is an important item in the case of children, or adults even, when the employment of a remedy is demanded for a period of time. There is every reason for presenting our medicines in as palatable and pleasant a form as possible. In addition, there are many persons of a nervous, delicate organization, particularly females, who cannot take the ordinary preparations of iron. They disorder the stomach—in their language are too heating—and thus not only fail to be assimilated, but, by perverting the gastric and intestinal secretion, seriously interfere with the digestion. Hence, instead of enriching the blood by new materials, we are merely cutting off the original supply, imperfect as it is, and making the gastric surfaces a centre of morbid irritation. We observe a similar but more complete abeyance of the nutritive functions, in most patients much reduced by an exhausting disease. However much iron may be indicated, it cannot be borne, much less appropriated by the absorbents, until the disgestion is restored by bitters and stimulants.

A marked peculiarity in the pyrophosphate of iron is the fact that it will, scarcely ever, in any of these cases disagree, and very frequently patients who cannot tolerate the ordinary forms of iron, will bear this well, and receive great benefit from its use. Like the others, it may fail to add to the blood a richer pabulum, from some fault in the vital processes of nutrition; yet unlike these, it will not aggravate the disorder for the relief of which it was given. Where the digestive powers are unimpaired, it matters little what preparation is selected, as far as its tonic action on the blood is concerned; since all—certain chemical reasons to the contrary notwithstanding—will fulfil this indication satisfactorily.—The new salt will supply the iron to the blood-globules as promptly, but not more so, than the others.

It has, however, another and more important property, which has entirely escaped attention; that adds new virtues to the iron and bestows on this special compound advantages possessed by none other in the Materia Medica. These arise from the pyrophosphoric acid. This acid, or the element, phosphorus—which, has not been definitely determined by chemists—exists alone in a free state in the great ganglionic nervous centres. According to Frémy, the phosphorus is combined with the brain-fat, forming what he calls the oleophosphoric acid. This, by boiling for some time in alcohol or water, splits up into olein and phosphoric acid. This brain-fat, cerebric acid or cerebrin, is the protein-body of the nerve-centres, and its great peculiarity is the amount of phosphorus it contains. Frémy makes it 9 parts to 1000. There are many other protein-bodies with specific properties that affect peculiar vital transformations, such as, for example, the nitrogenized element in the gastric juice, the pancreatic secretion, casein and the albumen of eggs or blood. This nitrogenized element, by its presence, without any chemical union with the other ingredients, forms a great diversity of new compounds from the same plasma; as, for example, the various secretions from the same blood.

It is, at the present time, tolerably well established (Virchow, Kölliker, Bennett) that all life starts from and is sustained by the agency of cell-growth, and that even morbid actions form no exceptions, but are carried on by the same organic forces. These cells, in their walls, nuclei and contents, contain a peculiar and distinctive protein-body; and being distributed universally in the solids and fluids of the body, are the great vital factors—the pervading life-force—by which organic functions are manifested. Formerly, it was thought that the peculiarities of this nitrogenized, albumenoid substance or protein-body, were due to the nitrogen in its composition, but it has since been found that phosphorus and sulphur are usually present. Both of these are contained in the albumen of the egg or of the blood, and in the casein of milk—fluids that contain every element necessary for the perfect development of living creatures. Milk is the most perfect type of the various constituents and their proportions that should exist in our diet.

| Nitrogenized elements in Albumen (Mulder). | | Nitrogenized elements in Casein. | |
|---|---|---|---|
| Carbon | 52.97 | Carbon | 53.61 |
| Hydrogen | 6.81 | Hydrogen | 7.11 |
| Nitrogen | 15.11 | Nitrogen | 15.47 |
| Oxygen | 23.54 | Oxygen | 17.99 |
| Sulphur | 1.57 | Sulphur | 1.11 |
| Phosphorus | .40 | Phosphorus | .74 |

It thus appears evident that phosphorus holds an important place amongst the other element that contribute to cell-life and nerve-power; but as all nitrogenized food contains it in the same proportions as it exists in the human organism, we cannot select a better remedy for defective nutrition than these ready-formed products. We should naturally suppose, what I have found by experience, that animal food would be the proper agent to restore flesh to an emaciated patient, since it contains each constituent necessary to its formation. At least, I have not found the phosphoric acid to add anything to the iron in such cases. Phosphoric acid united to a base—lime—exists in all the fluids and solids of the body. The phosphate of lime is formed in the vegetable from the elements in the soil; whence we derive it directly, or secondarily, through animals who have fed upon them. The phosphatic salts are received as such, and are probably carried through the blood to the solids, particularly to the bones, without suffering decomposition and re-construction; excepting, perhaps, in a small ratio. It scarcely could be requisite, when the supply presented to the stomach is always so abundant, to administer any phosphatic salt for the purpose of adding the phosphate of lime to the blood and thence to the bones. The defect of this saline in the bones is not due to the lack of the elements in the food, but to a fault in nutrition. When this is obviated, the common articles of diet will supply all the materials required. We conclude, therefore, that phosphorus is not demanded medicinally to build up the nitrogenized tissues of our bodies, nor are the phosphates to form the bones, since they are all presented to the blood in great abundance.

Phosphorus is regarded by therapeutical writers as a cerebral stimulant, exalting nerve-power directly, but the action of the heart indirectly, and only a moderate degree beyond the normal tension. Of all the organs, the reproductive are most sensibly affected; a fact satisfactorily accounted for in the male by our knowledge that the semen contains, according to Kölliker, over two per cent. of a phosphoretted fat. As throughout nature nothing is without a use, and every element has an importance, though we may fail to discover it; so we may safely conclude that phosphorus must exist in the nervous centres and the spermatic fluid as an integral constituent in their chemical composition. Probably it plays an important part in the normal excitability, and is intimately connected with the manifestation of mind, and the generation of the nervous influence.

In many conditions occuring in disease there might be a lack of this constituent, in a due proportion; precisely as there is of iron in anæmic states of the blood, when our only resource would be to present it in some assimilable form to the system, as there are no substitutes for the elementary bodies. In the case of phosphorus, here has always lain the difficulty; undergoing a slow oxidation or combustion at ordinary temperatures, even when floating on water, its substance would be burnt in the stomach, and a small particle adhering to the mucous surface would occasion irritation or inflammation. It could not be absorbed as phosphorus, and could only be remedial by the phosphorous and phosphoric acids that are formed. These would undoubtedly combine in the stomach with the earthy or alkaline bases, and be reduced to the state of the phosphates existing in the food. These, we know, suffer but little change in the blood, being found unchanged in all the solids and fluids, but particularly in the bones. From them, however, in normal, healthy nutrition, the phosphoric acid in the nerve-centres must be derived. Should there be a great depression of vital power, the acid is not liberated from its combination, in the same manner, as, we know, the iron is not, from the materials for digestion. The iron set free by assimilation in the blood is appropriated by the hæmatin; the phosphorus by the brain-fat. In hydræmia we give the iron in an easily assimilated form; one that does not tax the vital powers in separating it from a chemical combination, and straightway the blood begins to regain its color, and strength and vigor are infused into every organ. When a certain stage of recuperation has been attained, as shown by a more florid blood and a stronger pulse, the iron will be readily appropriated from the food, which, normally, is the source whence it is always obtained. The fault, originally, lay not in the absence of iron in the substances presented to the blood, but in an imperfect elaborating power, which failed to assimilate it. In like manner, I think, phosphoric acid may, from the same defect, not be separated from its compounds, and thus the ganglionic nervous centres be wanting in their normal stimulus. Hence would arise many nervous and neuralgic diseases, and nervous complications in many forms of debility. It is necessary for us to pass the phosphoric acid into the blood. This we can only do by giving it in a saline state, with a base that would be assimilated, and thus set it free. This is accomplished by the iron, which we know, in ordinary medicinal doses, is used in the blood; in other words, is appropriated by the hæmatin, and cannot be detected by any tests. It is a natural constituent in the red globules, and, consequently, not being foreign to the body, behaves precisely as any of the other elementary principles that form its structure. Strictly speaking, it is a food, and must be supplied as much as starch, sugar, oils and flesh. * *

We have employed the citro-ammoniacal pyrophosphate of iron, in certain conditions, with the most marked and gratifying results.

Whenever the blood becomes thin and watery, there are, almost invariably, troublesome attendant symptoms, seriously retarding the restoration of the patient to health. In all, there will be a lack of nerve-power, from the hydræmic state of the circulation. Hence, could we temporarily augment the stimulating properties of the blood, whilst we are administering the iron, we should prepare the way and present the conditions required for its assimilation, which otherwise might be impossible. Experience has taught most physicians this practical fact, and the indications have usually been fulfilled by the simultaneous use of wine and iron. We have found the pyrophosphate singularly appropriate under these circumstances, and as superior as a natural excitant must ever be over any substitute we may devise. Persons who have been overworked by mental application and prostrated by disquietude and care, or persons who have a shattered nerve-power from some constant source of bodily suffering, have a thousand anomalous symptoms dependant on an imperfectly generated and distributed nerve-power—such as wakefulness, trembling spasmodic movements, palpitations, etc. For this class of symptoms, the pyrophosphate of iron often affords relief in two or three days ; and thus prepares the way for the ultimate cure that may be expected from the martial salts. Many times, patients have expressed wonder at the calming and tranquilizing effects of the medicine; not only in mere functional aberrations and irregularities, but also in cases where actual disease existed in the nerve-centres. In both instances, the stimulation is immediate and transient, and can be of no avail, except by removing irregular nervous distribution; whilst the iron is appropriated more readily by the organic forces now freed from a great source of disorder. A lady in this city, with spinal meningitis in the cervical region, had great feebleness and trembling, but especially paroxysms of an asthmatic shortness of breath, that greatly interfered with the aëration of the blood. The first trial with this remedy removed, in a few days, the severity of the symptoms; so much so, that the patient was enabled to leave her bed. Her breathing was hurried only on exertion. The remedy becoming less potent in subsequent attacks, and then eventally quite useless, was abandoned, and other means were resorted to with the same ill success. The patient, after being under my care, without benefit, for three months, moved into the country, and nothing has been heard of her since. In other instances of anæmia, where time showed an organic basis for the nervousness, a temporary advantage has been gained by this form of iron; showing the stimulation afforded by it to the brain and spinal marrow. This stimulation, although only temporary, is of permanent value in all functional disorders of the nerve-power, where, in the mean time, we can rectify the states on which they are dependent. This is shown markedly in anæmia and chorea united. A young girl, 16 years of age, presented herself at the Hospital Clinique with symtoms of anæmia, amenorrhœa and chorea. She had been unwell at two periods, four months ago; but since then her turns had failed, and she had become affected by these involuntary motions, which now were so great and uncontrolable as nearly to forbid her standing or walking. She was ordered laxatives for the constipation, which was obstinate; and the pyrophosphate of iron in five grain doses, after each meal. This was the only treatment, from first to last. Her appetite, which had been capricious and uncertain, returned; the torpor of the bowels became less obstinate, and the involuntary jerkings of the muscles subsided to such a degree that the girl in two weeks walked to the Hospital unattended. In six weeks the menses returned, when the choreaic movements, which had become moderate, were greatly increased during their continuance. In the interval there was a continuous improvement, as the color of the face returned, but only a slight exacerbation during the next period. In three months the restoration to health was perfect. Perhaps the common forms of iron, as I have seen in two or three instances, might have been efficacious and attended with success, but I am confident the first stage of the cure would have been more tedious, from the constant muscular action which exhausts the patient. By the tranquilizing power of the phosphoric acid, the movements were moderated; the patient was enabled to fall asleep readily, which she could not do before, and the assimilation was strengthened, so that food and iron rapidly improved the blood.

In one other case of chorea and anæmic, the same happy result followed this course of treatment.

In palpitation of the heart in anæmic subjects, I have seen many instances of the power of this remedy in removing this symptom long before the blood was restored to its normal condition. But palpitation, when not due to impoverished blood entirely, may be, often times, equally amenable to this remedy.

A lady of this city, 45 years of age, feeble, emaciated, and the subject of paralysis agitans for many years, was tormented by an aggravated form of palpitation of the heart. This, at times, was very severe and persistent, and never benefitted by a variety of medicines prescribed by different physicians. The cause of this irregular and tumultuous action was evidently due to the defect in the nervous influence. The pyrophosphate of iron gave her the most prompt and perfect relief, so much so, that she sent a person affected with this disease to my office to obtain the same prescriptions. In this case, also, the result was equally satisfactory. In even more grave disorders of the heart, the value of this remedy has been signally shown.

A young gentleman, 23 years of age, came to my office, suffering fearfully from angina pectoris. He was first attacked five years previously, whilst in the country, and was obliged to give up his employment and travel for health. After six months' respite he obtained some relief, but from that time he had been followed by returns of the paroxysms at frequent intervals, though much less severe until of late, when they had become more grave than ever before. There was a sense of agony; a fear of impending death; and he was conscious of an irregular, tumultuous, labored throbbing of his heart, even in sleep, which was fitful, unrefreshing and disturbed by frightful dreams. His countenance was haggard and bespoke the utmost dejection, and he felt as though his chest was tightly bound by a chord, and as if each pulsation of his heart would be the last.

On examination, no organic disease could be discovered. The heart contracted tumultuously—by fits and starts—with a rolling, tumbling, uncertain action, but spasmodically, and with a sharp, metallic ring. The pause was irregular and intermittent, and the volume of blood in the artery was uncertain and unequal.

Latterly the young man had been employed in a wholesale dry goods store, in New York, and had passed most of the day on an underground floor. As he appeared to suffer from considerable gastric and hepatic disorder, two cathartic doses of blue mass were given, and followed by vegetable bitters and antacids. These failing, he was ordered assafœtida, valerian, camphor, etc., with the like ill result. At this time, two weeks from his first visit, he was much worse, so much so, that he fell on the floor insensible one afternoon after returning from business. He was now directed to take the pyrophosphate of iron, in five grain doses, three times a day, and to omit all other medicines whatsoever. The patient remained under the same circumstances as to air, diet, exercise, etc., and still persisted, from a fear of losing his situation, in going to the store every day. There was a sensible amelioration in the severity of his symptoms almost immediately; which became very apparent in four or five days. Insensibly he began to sleep with tolerable comfort, and to experience a more regular and equable action of the heart. At times, he would lose a sense of his condition, which ever had followed him like a malignant spirit. The change in his countenance, in the state of his pulse and rhythm of the heart, as discovered by auscultation, was remarkable. The improvement was steady but rapid, until the restoration to health was complete, which took place in four weeks, without any change in, or any addition to, the prescription. Now, after an interval of more than two years, he remains in perfect health, and was enabled to rush to arms, with thousands of other compatriots, for the defence of the old flag and the constitution of our fathers. He endured three months' service as readily as the others. Since he returned I found, on examination, that his heart acts perfectly and normally.

A young married woman, never pregnant, 34 years of age, was admitted into the Hospital a year since. She was thought by her physicians to have an aggravated, organic heart disease, that had nearly run its course. Her present illness dated back some eight months, and apparently commenced with gastric and hepatic disorder, which gradually induced despondency, nervousness, and palpitation of the heart. She had a sallow complexion, loaded tongue, vomiting, which was frequent, of a bitter matter; a strong, tumultuous and irregular action of the heart, with a metallic ring; a sense of great suffering and a fear of impending death. She was gloomy, hopeless, hysterical, with many shifting neuralgic pains.—Shortly before her admission she lost the use of the lower extremities—could not stand, though when in bed she could draw up her feet, but with great effort and difficulty. I could discover in her case no evidences of organic disease, and was inclined to think that when the functional disorders of the liver and digestive organs were corrected, it would be possible to mitigate, at least, the irregular action of the nervous system which was supposed to be mostly of a hysterical character. A variety of means, such as blue mass, bismuth, creosote, quinine and bitters were employed for two weeks or more, to correct the state of the stomach, check the vomiting, and restore the digestion—which objects were partially attained; but nerve-aberrations continued the same as at first. The pyrophosphate was now given conjointly with vegetable bitters and good diet. The same happy result followed as in the last case. Gradually the use of her limbs, and the regular, natural action of her heart were regained, though from the hysterical element in her case, the restoration to health was not as perfect or permanent as that of the young man just mentioned. She dwelt constantly on her troubles, and being much alone and neglectful of exercise, she relapsed, six months after dismissal, and was again treated in the same way, and in three weeks regained her usual health. During the present month I was called to her house and found her with the old symptoms, which she said had been coming on ever since the absence of her husband in the army. She requested the same medicine which had, the other times, been followed by such marked relief to the symptoms. Its power was equally apparent as before. For all the varied and anomalous symptoms of hysterical patients, which are usually some phase of irregular distribution of the nervous influence, the pyrophosphate acts with singular efficiency; diffusing and equalizing the nerve-power, and thus secondarily restoring a more active capillary circulation and a more healthful play of all the functions. Cases illustrative of this point are unnecessary in the milder forms of nervous disease, since the claims of our remedy are sufficientlyvindicated in the severer ones hitherto mentioned.

The pyrophosphate of iron has another property scarcely to be expected, and one we should never discover except by actual observation. All of the common preparations of iron are apt to oppress the stomach, coat the tongue and destroy the appetite, especially when the patient is much debilitated. Many, from a delicate, sensitive organization, cannot, under any circumstances, take iron with profit, it being, in their language, too heating. The pyrophosphate is friendly to the stomach, will never cause any irritation of the gastric surfaces, and, to our knowledge, has never disagreed with any patient, however incompatible the other forms may have been.—Besides, it appears to possess a tonic power, and will restore the appetite and digestion after the failure of bitters, quinine, wine, etc., often in extreme cases of anæmia, amenorrhœa and chlorosis, as we have witnessed in many instances in our obstetric clinique. It seemed to afford just the grade of stimulus required by the stomach, and the improvement, thus initiated, continued without interruption, under this single remedy, to the complete cure of the patient. This acceptability, friendliness, corrigent and roborant action of this form of iron on the digestive organs is a valuable peculiarity which renders it, in many persons and in many states of disease, superior to all others, and perhaps to any drug whatsoever.

Besides, its tastelessness, when dissolved in syrup, is a great recommendation in this age of sugar, when patients desire to die *sweetly*, and will not endure anything nauseous or unpleasant, though death be knocking at the door. This, we might expect in children who *bring up* their parents to a tolerably high state of discipline, and issue their orders of command from the cabinet councils of the nursery. We medical men, taking the world as we find it, are obliged to render our doses as palatable as possible for babies, both great and small. This object, without detriment in the choice of our means, is singularly and notably attained by the use of the syrup of the pyrophosphate of iron.

